# BMS-911543 inhibits viability of tumor and stromal cells and limits disease progression in genetically engineered mice with pancreatic cancer

**DOI:** 10.1186/2051-1426-2-S3-P186

**Published:** 2014-11-06

**Authors:** Thomas Mace, Reena Shakya, Benjamin Swanson, Thomas Ludwig, Omar Elnaggar, Hannah Komar, Jennifer Yang, Gregory Young, Wendy Frankel, Peter Muscarella, Tanios Bekaii-Saab, Mark Bloomston, Gregory Lesinski

**Affiliations:** 1 The Ohio State University, Columbus, OH, USA

## 

Pancreatic cancer (PCa) is resistant to cytotoxic therapies, and the profound immune suppressive nature of this disease renders patients non-responsive to immunologic therapies. Signaling downstream of IL-6 is important in the genesis and progression of PCa. The IL-6/Jak2/STAT3 axis also mediates expansion of myeloid-derived suppressor cells (MDSC). Indeed, the Jak2/STAT3 pathway is activated in human PCa specimens and cooperates with activated Kras to drive initiation and progression of PCa in murine models. We hypothesized that the Jak2 specific inhibitor (BMS-911543; Bristol-Myers Squibb) would elicit anti-tumor activity against PCa and decrease immune suppressive features of the disease. Treatment of *Kras *wild type (BxPC-3) and mutant (MIA Paca-2, Panc-1) human PCa cell lines *in vitro *with BMS-911543 decreased viability in a concentration-dependent manner. Similar inhibitory effects of BMS-911543 were also observed upon stromal-derived pancreatic stellate cells *in vitro*. These stromal cells have been previously shown by our group to rely on STAT3 signaling for survival, and secrete cytokines involved in MDSC generation. BMS-911543 also had immunomodulatory effects, as it significantly reduced IL-6/GM-CSF driven MDSC differentiation (HLA-DRlo CD11b+ CD33+) from healthy normal donor PBMC *in vitro*. To evaluate *in vivo *activity of BMS-911543, we used a genetically engineered PCa model with conditional expression of mutant *KrasG12D, tp53R270H*, and *Brca1 *alleles in pancreatic cancer cells (Brca1-KPC mice). This model accurately recapitulates many histologic and systemic manifestations of PCa observed in patients, but at an accelerated rate, which is advantageous for therapeutic studies. By 5 weeks of age, Brca1-KPC mice have adenocarcinoma with 100% penetrance, pSTAT3 in the tumor and stroma, high levels of MDSC, and increased plasma IL-6. At 5-6 weeks of age, mice (n = 5/group) were imaged by bioluminescent imaging (BLI) to confirm tumor burden and were then treated with vehicle or BMS-911543 by oral gavage daily for 14 days. Histologic analysis of pancreata from treated mice showed fewer foci of adenocarcinoma when compared to vehicle controls. Analysis of immune biomarkers will focus on Jak2/STAT3 driven processes such as the percentage of MDSC, T cell subset analysis and inflammatory cytokines both systemically and in the tumor microenvironment. These results provide rationale for the design of future clinical trials that target Jak/STAT signaling in patients with PCa.

